# The Peculiar Emergence of Mpox (Monkeypox): Directions for the Search for the Natural Reservoir and Vaccination Strategies

**DOI:** 10.3390/vaccines12101142

**Published:** 2024-10-04

**Authors:** Romulus Breban

**Affiliations:** Institut Pasteur, Unité d‘Epidémiologie des Maladies Emergentes, 75015 Paris, France; romulus.breban@pasteur.fr

**Keywords:** patient zero, disease invasion, epidemic emergence, mpox, monkeypox, animal reservoir, vaccination strategy

## Abstract

**Background/Objectives:** Mpox (monkeypox) is a zoonosis with origins in a currently unknown African reservoir. The first epidemiological accounts of mpox date back to the early 1980s, yet mpox only emerged as a pandemic threat in 2022–2023, more than 40 years later. This scenario is very different from those of other emerging diseases such as HIV and SARS, which immediately spread globally, in fully susceptible populations, starting from patients zero. **Methods:** We use mathematical modeling to illustrate the dynamics of mpox herd immunity in small communities in touch with the mpox natural reservoir. In particular, we employ an SEIR stochastic model. **Results:** The peculiar emergence of mpox can be explained by its relationship with smallpox, which was eradicated through universal mass vaccination in 1980. Mpox first emerged in small rural communities in touch with mpox’s animal reservoir and then spread globally. The relative isolation of these communities and their herd-immunity dynamics against mpox worked to delay the introduction of mpox in large urban centers. **Conclusions:** Mathematical modeling suggests that the search for the mpox animal reservoir would be most fruitful in communities with high mpox seroprevalence and small outbreaks. These are communities is tight contact with the mpox natural reservoir. We propose vaccinating individuals in communities in these communities to severely reduce the importation of cases elsewhere.

## 1. Introduction

In July 2022, a multi-country outbreak of mpox (monkeypox) was declared a Public Health Emergency of International Concern (PHEIC) as it spread rapidly via sexual contact across many countries where the virus appeared for the first time. The PHEIC was declared over in May 2023, following a sustained decline in global cases. However, mpox continued to spread. Recently, the WHO determined that the current upsurge in mpox in the Democratic Republic of the Congo (DRC) and a growing number of African countries also constitutes a PHEIC. Indeed, the origins of mpox are zoonotic, found in African regions where the disease remains endemic due to contacts with its animal reservoir. It appears that, through exportation of cases from endemic regions, mpox emerged as the most important Orthopoxvirus infection in humans, following the eradication of smallpox [1].

Over the past 50 years, the threat of emerging and re-emerging infectious diseases has steadily increased [2,3]. New emerging human pathogens have been identified [4,5], while outbreaks of emerging diseases became more frequent with time [6]. Some of the emerging infectious diseases have been Zika, SARS, COVID-19, HIV disease and 2009 H1N1 pandemic influenza. Their path to emergence is described using the concept of *patient zero*, the first disease case in a disease-naive community [7,8,9,10], who is expected to infect $R_{0}>1$ other individuals, either directly or through an intermediate vector. Then, each newly infected individual is expected to cause $R_{0}$ new infections, and so on. The subsequent epidemic dynamics are exponential and termed *disease invasion*. 

These dynamics, however, do not fit the emergence of mpox, which is peculiar due to its relationship to smallpox [11]. The first individual in a community to acquire mpox from the zoonotic reservoir would likely not find themself in a fully susceptible community. Since mpox and smallpox share cross immunity [12], smallpox herd immunity is protective against mpox infection [13]. Historically, smallpox was spread worldwide and its eradication was only achieved in 1980, through universal vaccination. Today, the vast majority of communities are not in touch with the mpox animal reservoir and still maintain herd immunity against mpox in the range of 7–60%, due to their smallpox epidemiology and history of vaccination [14,15]. Therefore, a case of mpox, resulting from contact with a zoonotic reservoir, does not fit the theory of patient zero. This is contrary to other emerging diseases, such as Zika [16,17], COVID-19 [18] and 2009 H1N1 pandemic influenza [19], where contact with the animal reservoir played only a very limited role, at the very beginning of the epidemic, in the introduction of patient zero.

The emerging dynamics of mpox depend on the dynamics of mpox herd immunity in the very same community. Herd immunity declines as immune individuals succumb to disease-unrelated causes. However, if the community is in contact with a mpox reservoir, then mpox outbreaks are expected to occur and, consequently, the mpox herd immunity is also expected to increase. Therefore, the ensuing dynamics of herd immunity may be complex. In this work, we discuss the dynamics of mpox herd immunity using empirical data and mathematical modeling based on a SEIR stochastic model; see refs. [20,21] and ([22], p. 19). We are particularly interested to describe typical rural communities in contact with mpox animal reservoirs. Hence, the typical epidemic threshold theory no longer holds, and the epidemic dynamics are generic, i.e., they remain approximately the same when the model is subject to arbitrary small perturbations. These features are not present in the traditional SEIR model. The overall goal of our modeling work is to provide a qualitative understanding of the epidemic dynamics and assist with open questions in the mpox epidemiology. In particular, we discuss the quest for identifying the mpox animal reservoir and strategies for vaccination against mpox.

## 2. Materials and Methods

It was recognized early that mpox is a zoonosis, where human cases can be explained by contact with animals, so-called *mpox introductions*, or other human cases [13]. To this day, the mpox natural reservoir remains largely unknown, although the main animal hosts are believed to be found among wild African rodents [23,24]. Mathematical models have been employed from the very beginning to provide insight in the epidemiology of mpox [13]. Meanwhile, a wealth of information on mpox has been gathered to inspire new modeling studies [11,25].

### 2.1. Mpox Herd Immunity in Data

During 1980–1984, soon after the eradication of smallpox, mpox outbreaks were observed in DRC communities, in what might be the first epidemiological account on mpox [13]. It was found that mpox outbreaks were self-limiting, in the context where herd immunity was close to 85% in 1980. Mpox thus began to emerge immediately following smallpox eradication, encountering high levels of herd immunity. Outbreaks were small and extinguished rapidly, yet new introductions constantly occurred, eventually leading, in the long term, to endemic states in certain communities [26]. The immune responses to mpox infection [27] are not fully understood. No validated correlate of immunity or protection after vaccination has been identified so far [28]. Furthermore, 37 cases of mpox reinfection were documented in nine countries between 11 May 2022 and 30 June 2023 [29]. However, reinfection does not appear prevalent considering the 54,722 laboratory-confirmed cases of mpox in the same 9 countries from 1 January 2022 through 30 November 2023 [30].

In epidemiology, Orthopoxvirus seroprevalence in humans is typically measured as an indicator of past contact with Orthopoxviruses and herd immunity to Orthopoxvirus infection. Measurements have been made in the general population of high-income countries [31,32], as well as remote rural communities [33,34,35,36,37,38,39]. Mpox endemic areas have been identified in Cameroon, the Central African Republic (CAR), the DRC and Nigeria [40]. In 2011–2012, Orthopoxvirus seroprevalence in DRC communities was 60% (95% confidence interval (CI) 53–65%), that is, as high as 96% (95% CI 91–99%) among individuals vaccinated against smallpox and as low as 26% (95% CI 18–35%) among unvaccinated individuals, suggesting regular contact by DRC communities with Orthopoxviruses [41]. During 2001–2021, Orthopoxvirus seroprevalence in populations experiencing mpox outbreaks in the CAR was, on average, 55% (95% CI 49–61%), suggesting regular contact by CAR communities with Orthopoxviruses [39] as well.

### 2.2. Mathematical Modeling of Mpox Transmission and Herd Immunity

To model a typical rural community in touch with a mpox reservoir, we propose a continuous-time Markov chain of type *Susceptible–Exposed–Infectious–Recovered* (SEIR). This framework requires the reasonable assumption that mpox cases recover with lifelong immunity. We proceed by introducing the integer variables S, E, I, and R to represent the numbers of susceptible, exposed (i.e., infected but not yet infectious), infectious and recovered individuals; the total population is given by N=S+E+I+R. An auxiliary, herd immunity variable is defined as hi=R/N. The processes of the Markov chain are listed in Table 1 and its parameters are listed in Table 2.

We parameterized our SEIR Markov chain using calibrated values and values within the ranges listed in Table 2. The life expectancy at birth 1/μ was calibrated such that, in the absence of mpox introductions, herd immunity is expected to decline from 85% in 1980 to 10% in 2025, simply due to the disease-unrelated death of immune individuals. We used the expected community size in the absence of disease, $N_{0}=\pi /\mu$, and 1/μ to obtain π, the inflow of susceptible newborns. We used $R_{0}$ estimates to obtain the disease transmissibility β and the case–fatality ratio to obtain the rate of death due to disease, σ. The overall goal of our parameterization is to illustrate typical transmission dynamics of mpox in small communities. Our stochastic model was integrated numerically using the Gillespie direct method [43].

## 3. Results

The SEIR Markov chain is simple enough that several key analytical results can be obtained. They clearly and rigorously mark the difference between traditional SEIR models considering human-to-human transmission alone and SEIR models which also consider contact with a disease reservoir. Analytical results also provide a backbone to qualitatively understand numerical simulations.

### 3.1. Analytical Results

Important results can be obtained analytically for the case where β=0, i.e., if humans were dead-end hosts for mpox and human-to-human transmission did not occur. In this case, all the rates of the Markov chain are linear in the population variables and moment closure techniques [44,45] yield the following system of ordinary differential equations (ODEs):

(1) $$\begin{array}{c} \frac{d\langle S\rangle }{dt}=\pi -\delta \langle S\rangle -\mu \langle S\rangle \\ \frac{d\langle E\rangle }{dt}=\delta \langle S\rangle -\upsilon \langle E\rangle -\mu \langle E\rangle \\ \frac{d\langle I\rangle }{dt}=\upsilon \langle E\rangle -\gamma \langle I\rangle -\sigma \langle I\rangle -\mu \langle I\rangle \\ \frac{d\langle R\rangle }{dt}=\gamma \langle I\rangle -\mu \langle R\rangle \end{array}$$

where 〈·〉 denotes the average over all stochastic orbits as a function of time, i.e., the expected population numbers. The above ODE system has a unique equilibrium, representing a stable endemic state with components

(2) $${\langle S\rangle }_{*}=\frac{\pi }{\mu +\delta },{\langle E\rangle }_{*}=\frac{\delta {\langle S\rangle }_{*}}{\mu +\upsilon },{\langle I\rangle }_{*}=\frac{\upsilon {\langle E\rangle }_{*}}{\mu +\gamma +\sigma },{\langle R\rangle }_{*}=\frac{\gamma {\langle I\rangle }_{*}}{\mu }$$

Human-to-human transmission (i.e., β>0) makes this endemic state more severe, where the average numbers of exposed, infectious and recovered individuals increase, and the average number of susceptible individuals decreases. The uniqueness of the endemic state implies that there exists no disease-free state and epidemic threshold when the community is in touch with a disease reservoir, i.e., when δ>0.

If the community is large so that each population variable is large (i.e., order 1000 and larger), then the dynamics of the SEIR Markov chain can be approximated by the conventional SEIR ODE system, which here plays the role of *mean-field approximation* for the stochastic chain [44,45].

(3) $$\begin{array}{c} \frac{d\langle S\rangle }{dt}\approx \;\pi -\delta \langle S\rangle -\frac{\beta \langle S\rangle \langle I\rangle }{\langle N\rangle }-\mu \langle S\rangle \\ \frac{d\langle E\rangle }{dt}\approx \;\delta \langle S\rangle +\frac{\beta \langle S\rangle \langle I\rangle }{\langle N\rangle }-\upsilon \langle E\rangle -\mu \langle E\rangle \\ \frac{d\langle I\rangle }{dt}\approx \;\upsilon \langle E\rangle -\gamma \langle I\rangle -\sigma \langle I\rangle -\mu \langle I\rangle \\ \frac{d\langle R\rangle }{dt}\approx \;\gamma \langle I\rangle -\mu \langle R\rangle \end{array}$$

In this case, the fluctuations in the population variables are of the order square root of the corresponding expectations (i.e., the relative fluctuations are order 3% and smaller) and the stochastic dynamics are well approximated by the ODE system in Equation (3). The basic reproduction ratio of the SEIR model is defined when there is no contact with disease reservoirs (i.e., δ=0) and is given by [46].

(4) $$R_{0}=\frac{\beta \upsilon }{\left(\mu +\upsilon \right)\left(\mu +\gamma +\sigma \right)}$$

### 3.2. Numerical Results

To illustrate the epidemiological dynamics of mpox, we simulated outbreaks taking place in a rural community in touch with the zoonotic reservoir of mpox. The initial condition was chosen to model the epidemiological setup starting with 1980, just after the eradication of smallpox: $S\left(0\right)=\left(1-hi\left(0\right)\right)N\left(0\right)$, $E\left(0\right)=0$, $I\left(0\right)=0$, and $R\left(0\right)=hi\left(0\right)N\left(0\right)$. In particular, the mpox herd immunity at time zero was considered to be $hi\left(0\right)=85\%$, assuming that all individuals in the community were vaccinated against smallpox before 1980 [11]. The contact rate with the mpox reservoir, δ, was varied to distinguish various epidemiological scenarios.

If δ=0 years^−1^, then the community is not in touch with the disease reservoir. The individuals with acquired immunity against mpox are steadily replaced by susceptible individuals who are no longer exposed to disease. Hence, the herd immunity wanes steadily down toward zero, while no epidemic outbreak takes place. This fits the smallpox/mpox epidemiology in communities of countries where mpox is not endemic. The communities in such countries are not in touch with the mpox reservoir and still maintain low levels of herd immunity against mpox due to their smallpox epidemiology.

**Figure 1:**
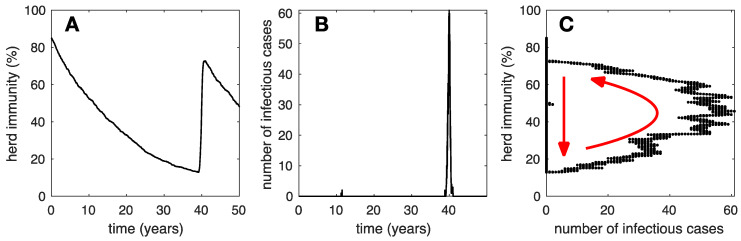
Typical epidemic dynamics in a community in weak contact (i.e., δ=0.0001) with the mpox reservoir. We illustrate (**A**) herd immunity versus time, (**B**) number if infectious individuals versus time, and (**C**) herd immunity versus the number of infectious cases. Initially, herd immunity is high and declines toward zero (vertical red arrow), until an mpox outbreak occurs and herd immunity reaches high values, again (curved red arrow).

In Figure 1, we illustrate the case where δ is only slightly larger than zero (i.e., δ=0.0001 years^−1^). In Figure 1A, the simulated herd immunity, hi, decreases steadily with time until, due to a mpox introduction, an outbreak takes place and causes hi to increase. Furthermore, during the outbreak, $I\left(t\right)$ undergoes typical peaked dynamics; see Figure 1B. However, mpox introductions from the zoonotic reservoir into the rural community occur very rarely, as the number of susceptible individuals increases and the population-level rate of mpox introductions, δS, becomes substantially larger than zero. The maximum rate can be estimated as $\delta N_{0}=0.1$ years^−1^. Thus, one mpox introduction is expected in the simulated rural community no sooner than once every 10 years. At the time of the introduction, a significant fraction of individuals in the community may be susceptible, resulting in a large outbreak.

If no mpox zoonotic introductions occur during an outbreak, then the outbreak is due to human-to-human disease transmission and goes extinct due to the depletion of susceptible individuals. The mpox introduction causing the outbreak is similar to a patient zero causing an epidemic in a community with herd immunity. The expected number of secondary cases caused by the mpox introduction is the reproduction number $R^{\prime }=\left(1-hi\left(t_{1}\right)\right)R_{0}$, where $t_{1}$ is the time of the mpox introduction.

In Figure 1C, we illustrate the dynamics of herd immunity, hi, versus those of the number of infectious cases, I. At the beginning, I=0 and the dynamics follow the vertical downward arrow. Then, a large outbreak takes place and the dynamics follow the curved arrow back to large values of hi. The dynamics are thus recurrent, where each outbreak pushes herd immunity back toward larger values.

**Figure 2:**
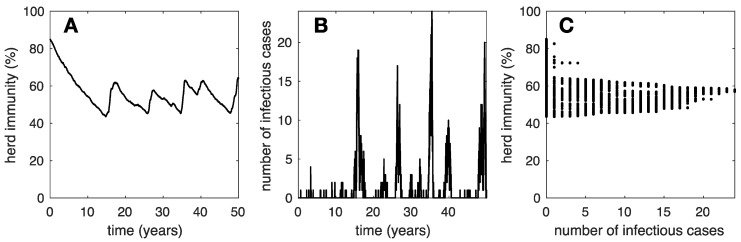
Typical epidemic dynamics in a community in moderate contact (i.e., δ=0.003) with the mpox reservoir. We illustrate (**A**) herd immunity versus time, (**B**) number if infectious individuals versus time, and (**C**) herd immunity versus the number of infectious cases. Note that recurrent outbreaks maintain the herd immunity above 40%.

Increasing the contact rate of the individuals in the rural community with the zoonotic reservoir by a factor of 30 (i.e., δ=0.003 years^−1^) yields a very different epidemiological picture. Smaller and more frequent outbreaks can be discerned; see Figure 2. It is also apparent that several introductions may occur during a major outbreak. Hence, in this case, the dynamics of patient zero do not apply, since they assume that the whole outbreak is due to a single introduction. Furthermore, Figure 2C shows that herd immunity varies only within a narrow interval (i.e., 40–65%), compared with the dynamics in Figure 1C (i.e., 15–75%), where mpox introductions are very rare, essentially one per outbreak.

**Figure 3:**
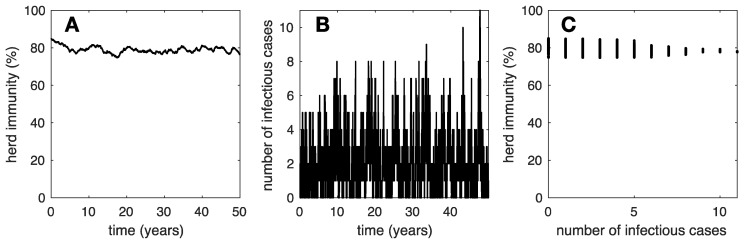
Typical epidemic dynamics in a community in strong contact (i.e., δ=0.1) with the mpox reservoir. We illustrate (**A**) herd immunity versus time, (**B**) number if infectious individuals versus time, and (**C**) herd immunity versus the number of infectious cases. It this case, the dynamics may be thought as an endemic state.

Further increasing the contact rate δ in our simulations, to the point where each susceptible individual in the rural community is expected to become a mpox introduction within 10 years (i.e., δ=0.1 years^−1^), we obtain the results illustrated in Figure 3. Figure 3A,C show that herd immunity remains in the vicinity of 80%. Figure 3B shows that the outbreaks are even smaller and more numerous, and the number of infectious cases amounts to an approximate average of 2.4 infected individuals out of nearly 1000 community members at any time.

## 4. Discussion

Mathematical modeling can provide an illustration of the peculiar emergence of mpox and assistance with questions about mpox epidemiology. Here, we focused on the mpox epidemiology at the community level, in endemic settings. A major question is to confirm the zoonotic origins of mpox and identify the animal reservoir. Currently, the same mpox strain, whether clade 1 or 2, has never been identified in an animal–human pair in endemic settings [47] to confirm the zoonotic transmission of mpox. Furthermore, live mpox virus has only rarely been isolated in wild animals: a squirrel (*Funisciurus anerythrus*) in the DRC in 1985, a sooty mangabey (*Cercocebus atys*) in Ivory Coast in 1992 [23,48] and, more recently, another squirrel (*Funisciurus bayonii*) and two rats (*Stochomys longicaudatus*, *Cricetomys* sp.) [49]. Although contact with terrestrial rodents (*Cricetomys* and *Graphiurus*) and arboreal rope squirrels (*Funisciurus*) is believed to explain most mpox introductions in human populations [23], the mpox reservoir remains unidentified. A recent study [50] proposes that the most probable mpox reservoir is *Funisciurus anerythrus*. It should be noted that the mpox reservoir is expected to change with human population growth, deforestation, climate change, etc.

Clues to identifying the mpox reservoir may be found among the human activities in rural communities where mpox remains endemic, which are presumably in touch with the mpox reservoir. In August 2022, the WHO updated their guidelines for establishing mpox surveillance systems [51] and thorough analyses followed [52,53]. Although one single mpox case already constitutes an outbreak, outbreak investigation favors large outbreaks. Even so, large outbreaks may not be fully identified by the surveillance protocols and small outbreaks may even go unnoticed. This is quite significant because, according to our simulation results, a community experiencing large mpox outbreaks is not in tight contact with the mpox reservoir (Figure 1). Thus, identifying the reservoir may be particularly challenging if investigations are devoted only to those communities. In contrast, we propose that investigations should be devoted to communities with elevated herd immunity and small, frequent outbreaks (Figure 3), which may even go unreported, as mpox cases may be considered common occurrence by the inhabitants of such communities. Identifying the natural reservoir for mpox remains subject to complex investigations [54,55,56] with major benefits for prevention through One Health approaches.

The global mpox epidemiology remains of great concern. In December 2023, the WHO assessed the mpox risk in the long term as being low for the general population in countries not affected prior to the current outbreak [57]; see Ref. [58] for further discussion. However, the risk remains moderate for high-risk groups (i.e., people likely to be exposed and their contacts) and the general population in countries with historical mpox transmission and their neighbors. Most importantly, the risk is high for the general population in the DRC, a country where 11 out of 26 administrative regions are declared mpox endemic areas. See Ref. [59] for an epidemiological update of the outbreak of mpox in the DRC.

Effective vaccination strategies are needed to protect vulnerable populations and break the importation of mpox cases from endemic regions, restricting the circulation of mpox. Many publications [60,61,62] have analyzed the parameters of the current mpox vaccines and proposed prioritizing vaccination for professionals and ring vaccination in the highest-risk group. The WHO published similar guidelines for mpox vaccination in November 2022 [62]. Many high-income countries stockpiled vaccines [63] and implemented national mpox vaccination programs for high-risk groups, with the explicit strategy to protect the most vulnerable populations. However, other vaccination strategies may be beneficial, as well. They can be run in parallel for additional epidemiological benefits.

The mpox epidemiology is heterogeneous across communities. Most communities are not in touch with the mpox reservoir and still maintain low levels of herd immunity against mpox due to their smallpox epidemiology and history of vaccination. Only recently have some of these communities imported mpox cases whose transmission chains trace back to the mpox endemic areas. A different mpox vaccination strategy would be to vaccinate individuals community by community, depending on the mpox epidemiology in each community.

It is assumed that the relatively few communities in touch with the mpox zoonotic reservoir are rural communities located within mpox endemic areas. Furthermore, these communities may have much larger herd immunity than others [39,41], actually stalling the emergence of mpox. The exportation of cases may be considered relatively low, owing to limited traffic between communities. The phenomenon whereby population clustering within communities stalls epidemic spread is known as *population viscosity* [64] and has been noted to a lesser extent for other zoonotic pathogens such as SARS [65], rabies [66], H5N1 avian influenza [67] and simian immunodeficiency virus [68].

The epidemiology of the community structure prevented, from 1980 until very recently, extended worldwide mpox outbreaks. To reinforce mpox immunity in the community structure and increase population viscosity, we propose a *survivor bias* vaccination strategy. That is, the community that survived in tightest contact with the mpox reservoir gets vaccinated with priority. This community may be recognized by its large Orthopoxvirus seroprevalence and small mpox outbreaks; knowledge of the mpox animal reservoir is not needed. However, estimates of the Orthopoxvirus seroprevalence and tight surveillance are needed. Further increasing mpox herd immunity in these communities using vaccination will lead to further protection for neighboring communities from human mpox introductions.

Many high-income countries have developed mpox vaccination programs and successfully secured the vaccine doses to run these programs, while African countries still struggle to perform this [69]. However, African countries may require an even greater public health effort to curb their mpox epidemics: mpox vaccination campaigns. A mpox vaccination campaign is scheduled to start on 2 October in the DRC, targeting only adults (i.e., healthcare professionals, park rangers and sex workers) in six DRC administrative regions. To this end, the United States and the European Union donated about 265,000 mpox vaccine doses to the DRC [70]; Japan also offered to contribute [71]. Another mpox vaccination campaign is currently unfolding in Rwanda, targeting high-risk individuals in seven administrative regions that border the DRC [72,73].

While current mpox vaccination campaigns target certain (not all) mpox endemic regions, more can be implemented for vaccination to reduce the number of imported mpox cases. A large-scale Orthopoxvirus seroprevalence study may be run in parallel to detect communities where the seroprevalence is large. Then, in the spirit of the survivor bias strategy, we propose priority vaccination for the communities where the seroprevalence exceeds a given threshold.

## 5. Conclusions

Already in the early 1980s, epidemiological accounts in the DRC suggested that mpox could eventually emerge to fill the niche left open by the eradication of smallpox. Mathematical modeling can provide qualitatively robust insights into key questions of mpox epidemiology. First, modeling suggests that the mpox animal reservoir is most easily identified in communities with large herd immunity and small, frequent mpox outbreaks. Second, for the question of how to prioritize vaccination to prevent the worldwide importation of mpox cases, modeling suggests vaccinating with priority the communities in closest contact with the mpox reservoir. This insight may be integrated within mpox vaccination campaigns in endemic countries to expand their strategic objectives.

## Figures and Tables

**Table 1:** Stochastic processes of the SEIR Markov chain and their corresponding rates.

Process	Definition	Rate
Inflow of susceptible individuals	S→S+1	π
Disease-unrelated death of susceptible individuals	S→S−1	μS
Mpox infection from the reservoir	S→S−1,E→E+1	δS
Human-to-human transmission	S→S−1,E→E+1	βSI/N
Exposed human becomes infectious	E→E−1,I→I+1	νE
Disease-unrelated death of exposed individuals	E→E−1	μE
Recovery of infectious individuals	I→I−1,R→R+1	γI
Disease-induced death of infectious individuals	I→I−1	σI
Disease-unrelated death of infectious individuals	I→I−1	μI
Disease-unrelated death of recovered individuals	R→R−1	μR

**Table 2:** Parameters of the mpox disease within the SEIR framework. The life expectancy at birth 1/μ is calibrated such that, in the absence of mpox introductions, the expected herd immunity declines from 85% in 1980 to 10% in 2025. The parameter π is estimated from the size of the disease-free community and the life expectancy at birth. The parameter σ is obtained from the case–fatality ratio. The transmissibility parameter β is obtained using Equation (4), $R_{0}$ estimates, and estimates for the rates which occur in the right-hand side of Equation (4).

Parameter	Symbol	Range	Simulation Values	Ref.
Inflow rate of susceptible individuals	π		47.62 years^−1^	
Life expectancy at birth	1/μ		21 years	
Size of the disease-free community	$N_{0}=\pi /\mu$	300–10,000	1000	
Rate of contact with the reservoir	δ		0.0001 years^−1^, 0.003 years^−1^, 0.1 years^−1^	
Mpox transmissibility	β		37.60 years^−1^	
Basic reproduction ratio	$R_{0}$	1.46–2.67	2.07	[11]
Incubation/latent period	1/ν	3–20 days	0.038 years	[42]
Infectiousness period	1/γ	2–4 weeks	0.058 years	[42]
Disease-induced death rate	σ		0.817 years^−1^	
Case–fatality ratio	$\sigma /\left(\sigma +\gamma \right)$	0.1–10%	4.5%	[42]

## Data Availability

Data is contained within the article.
